# Preferential Duplication of Conserved Proteins in Eukaryotic Genomes

**DOI:** 10.1371/journal.pbio.0020055

**Published:** 2004-03-16

**Authors:** Jerel C Davis, Dmitri A Petrov

**Affiliations:** **1**Department of Biological Sciences, Stanford UniversityStanford, CaliforniaUnited States of America

## Abstract

A central goal in genome biology is to understand the origin and maintenance of genic diversity. Over evolutionary time, each gene's contribution to the genic content of an organism depends not only on its probability of long-term survival, but also on its propensity to generate duplicates that are themselves capable of long-term survival. In this study we investigate which types of genes are likely to generate functional and persistent duplicates. We demonstrate that genes that have generated duplicates in the *C. elegans* and S. cerevisiae genomes were 25%–50% more constrained prior to duplication than the genes that failed to leave duplicates. We further show that conserved genes have been consistently prolific in generating duplicates for hundreds of millions of years in these two species. These findings reveal one way in which gene duplication shapes the content of eukaryotic genomes. Our finding that the set of duplicate genes is biased has important implications for genome-scale studies.

## Abstract 2

Gene duplication is a key process in genome evolution. These authors show that highly conserved genes duplicate more often and are therefore likely to contribute more to the content of eukaryotic genomes

## Introduction

Gene duplication is the most important source of new genes and consequently a vital source of genetic novelty (Ohno 1970). Recently, the availability of completely sequenced genomes has sparked renewed attention in this subject at the genome scale. Most genomic studies of gene duplication have focused on the mechanisms responsible for generating duplicate genes, the consequences of gene duplication for genetic redundancy, or the effect that duplication has on the molecular evolution of the genes involved (Seoighe and Wolfe 1999; Lynch and Conery 2000; Dermitzakis and Clark 2001; Van de Peer et al. 2001; Gu et al. 2002, Gu et al. 2003; Kitami and Nadeau 2002; Kondrashov et al. 2002; Nembaware et al. 2002). Comparatively less attention has been devoted to the essential question of whether some genes are more likely to give rise to functional and persistent duplicates than others and thus contribute more to the gene content of eukaryotic genomes (but see Kondrashov et al. 2002; Nembaware et al. 2002).

Investigating this aspect of gene duplication will not only help answer questions about gene content—such as why certain proteins duplicate to generate multigene families while others remain in single copy—but will provide insight into the process of duplication itself. Each of the three steps leading to the generation of preserved gene duplicates, including their (1) mutational generation, (2) fixation in a population, and (3) preservation through a period when they may be functionally redundant, may favor some genes over others. For example, gene duplicates that lead to an advantageous increase in gene dosage will be preferentially fixed by positive selection, as has been observed in bacteria and Saccharomyces cerevisiae (Romero and Palacios 1997; Brown et al. 1998; Dunham et al. 2002). For other genes, for which stoichiometry is important, the converse may be true: gene duplication may be strongly deleterious (Gerik et al. 1997), and while such duplications may commonly arise in single individuals, they are unlikely to become fixed in the population.

The step of preservation also has a great potential to create a bias in the types of genes that duplicate since the vast majority of duplicate gene copies that arise in a population are rapidly lost to nonfunctionalizing mutations (Lynch and Conery 2000). Theoretical accounts of duplicate gene preservation make various predictions about the types of genes that will be preserved following duplication. Specifically, these models predict that genes with a larger number of *cis*-regulatory regions, expressed in many tissues (Lynch et al. 2001) or encoding multidomain proteins (Gibson and Spring 1998; Stoltzfus 1999), will be preferentially preserved. By investigating the molecular attributes of the types of genes that duplicate, we may be able to validate these predictions and determine which steps in the process of duplication act as a selective sieve, promoting the duplication of some genes and hindering the duplication of others.

Beyond providing information about the mechanisms of duplication, data about the biases in which genes duplicate will serve as an essential baseline for other genome-scale studies in this field. For example, recent work has argued that gene duplication leads to a relaxation of selection and consequently an elevation in the rate of molecular evolution for the duplicated genes (Kondrashov et al. 2002; Nembaware et al. 2002). In support of this argument, these studies compared the evolutionary rate of genes that had duplicated to the rate of genes that were in single copy. A higher rate of evolution for the genes with duplicates was taken to support their hypothesis. One problem with this approach is that it is based on the assumption that the set of genes that generate duplicates is not biased with respect to the genes' rate of evolution. Indeed, if the genes that duplicate had higher rates of evolution prior to duplication, this would invalidate the above conclusions. Similarly, any study that reveals differences between the properties of duplicate genes and those in single copy (Kondrashov et al. 2002; Nembaware et al. 2002; Gu et al. 2003) should hesitate to conclude that these differences are caused by duplication per se without considering the biases in the attributes of the genes that lead to duplicates. In some cases, the authors themselves acknowledge this problem (e.g., Kondrashov et al. 2002; Gu 2003).

For these reasons we chose to investigate a bias in the molecular attributes of the genes that duplicate. One very informative gene attribute is the rate of protein evolution defined as the number of nonsynonymous substitutions per nonsynonymous site in a given time (*K*_A_). This measure of protein evolution has been shown to be related to several important properties of genes, including dispensability, level of expression, and the number of protein–protein interactions (Hirsh and Fraser 2001; Pal et al. 2001; Fraser et al. 2002). We chose to compare the rates of evolution of the genes that have given rise to observable duplicates in the well-studied genomes of S. cerevisiae and Caenorhabditis elegans with those that have not.

**Figure 1:**
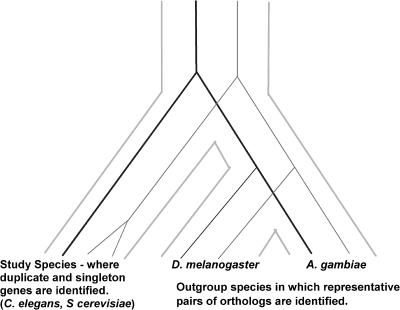
The Approach Used to Estimate the Rate of Evolution for Duplicate and Singleton Genes For each duplicate (gray lines) and singleton (black lines) gene/pair in S. cerevisiae and C. elegans, unduplicated orthologs were identified in D. melanogaster and A. gambiae. The *K*_A_ between this representative pair of orthologs was taken as an estimate of the rate of evolution of duplicate and singleton genes in the study species that is independent of the effects of duplication on molecular evolution.

Such a comparison is not straightforward since gene duplication itself may affect the rate of molecular evolution (Lynch and Conery 2000; Kondrashov et al. 2002). To avoid this problem, we did not use the rate of evolution of each singleton and duplicate pair in S. cerevisiae and C. elegans (the “study genes”), but instead measured evolutionary rates in two distantly related outgroup species, Drosophila melanogaster and Anopheles gambiae (such a pair of orthologs is referred to as a “representative pair”). Because evolutionary rates for a particular gene are highly correlated in diverse lineages (Bromham and Penny 2003), we reasoned that the nonsynonymous divergence between the members of each representative pair would be a good proxy for the rate of evolution of the study genes in a way that is unaffected by the process of duplication (Figure 1). Our results reveal that the genes that have duplicated in the genomes of S. cerevisiae and C. elegans appear to be a biased set of slowly evolving genes and that slowly evolving genes have been consistently prolific in generating duplicates for hundreds of millions of years in these lineages.

## Results

### Evolutionary Rates of Duplicate and Singleton Genes

**Figure 2:**
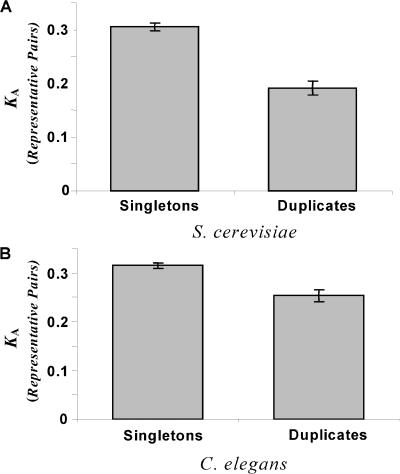
A Comparison of the Evolutionary Rates of Duplicate and Singleton Genes The average rate of nonsynonymous evolution (*K*_A_) for representative pairs of duplicate and singleton genes in the two study organisms S. cerevisiae (A) and C. elegans (B) is shown. Representative pairs of duplicate genes evolve significantly more slowly in both study organisms (Mann–Whitney U test, *p* < 0.001).

The number of duplicate pairs and singleton genes identified in the genomes of S. cerevisiae and C. elegans and the number of representative pairs of these genes found in D. melanogaster and A. gambiae are provided in Table 1. Our comparison of the nonsynonymous divergence between orthologs of these two classes of genes revealed that representative pairs of duplicates in both S. cerevisiae and C. elegans have much slower rates of evolution (Mann–Whitney U test, *p* < 0.001 for both) (Figure 2). The representative pairs of the duplicated genes in S. cerevisiae evolve on average more than 50% slower than the representative pairs of singletons (0.192 versus 0.302), while in C. elegans the difference exceeds 25% (0.230 versus 0.296).

**Table 1:**
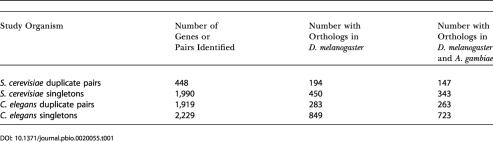
Number of Genes/Pairs Identified in the Study Organism and Number of Orthologs of These Genes Found in D. melanogaster and A. gambiae

**Figure 3:**
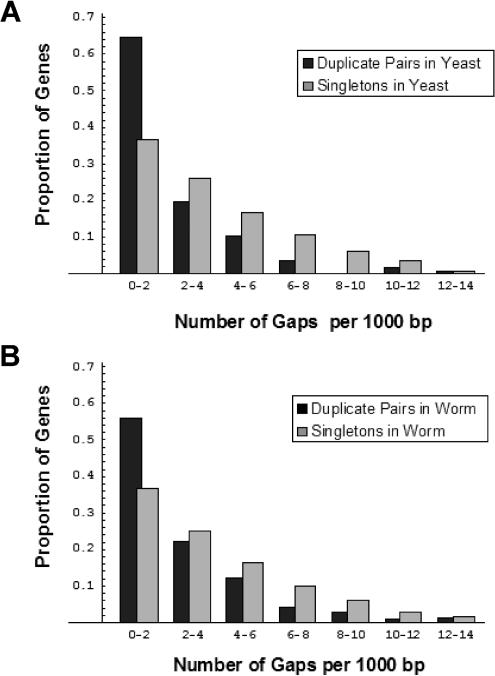
A Comparison of the Rate of Structural Evolution for Duplicate and Singleton Genes For each representative pair, the number of gaps per aligned nucleotide was calculated. For both S. cerevisiae (A) and C. elegans (B), representative pairs of duplicates have significantly fewer insertions per basepair than representative pairs for singletons (Mann–Whitney U test, *p* < 0.0001 for both).

In addition to estimating rates of evolution for representative pairs of the two classes of genes, we also attempted to quantify structural protein evolution by computing the number of gaps per basepair in the alignments of the representative pairs. We reasoned that this measure is likely to be a monotonic proxy for the number of indels that have occurred in the evolution of a protein since the split of A. gambiae and D. melanogaster. Results from this analysis echoed those of the *K*_A_ comparisons: representative pairs of duplicate genes in both S. cerevisiae and C. elegans are much less likely to have accumulated insertions or deletions than representative pairs of singletons (Mann–Whitney U test, *p* < 0.0001 for both) (Figure 3).

To further validate these conclusions, we wanted to test several potential sources of error in our analysis of both *K*_A_ and the indel rate. First, some of the orthologs identified in D. melanogaster and A. gambiae have undergone duplication in these lineages. This could both affect their rates of evolution, as discussed above, and also lead to the identification of the slowest evolving paralog in D. melanogaster and A. gambiae for the representative pairs of study genes. The latter effect can lead to an artificially low estimate of the evolutionary rates. To test for this possibility, we repeated our analysis using only representative pairs that have not duplicated in either D. melanogaster or A. gambiae. Although this analysis included substantially fewer genes (see Materials and Methods), the results remained unchanged and strongly statistically significant (Mann–Whitney U test, *p* < 0.005 for both organisms). Second, we wanted to make sure that the bias is not due to the peculiarly slow evolution of duplicate genes in multigene families. A reanalysis for only those duplicated genes (in the study organisms) with no other paralogs in the genome revealed very similar results (data not shown). Third, it is possible that our conservative definition of “singleton” may have artificially biased the set of singletons towards rapidly evolving genes. This could be true if slowly evolving singleton genes tend to possess anciently conserved, widely shared protein domains. By generating homology to other genes, these domains may make these singletons fall below the conservative *E*-value cutoff that we used. To test this possibility, we relaxed our criteria for singleton genes to include all those genes with no *E*-value less than 10^–10^. The average rate of evolution for this group of singleton genes was no different than for the former set (data not shown).

### Biased Mutation Cannot Explain the Lower *K*_A_ of Duplicates

The simplest interpretation of these data is that the genes generating preserved duplicates are a biased set of constrained, slowly evolving proteins. An alternative explanation is that representative pairs of singletons are found in genomic regions with a higher mutation rate than are representative pairs of duplicates—although there is no a priori reason why this should be true. One way of testing this possibility is to compare the number of synonymous nucleotide substitutions per synonymous site (*K*_S_) for the representative pairs of the two classes of genes. This measure is customarily used as a proxy for mutation rate because substitutions at synonymous sites are generally thought to be selectively neutral. However, in many genes, especially those expressed at high levels, synonymous sites appear to be under selection, as evidenced by codon bias. For such genes, the rate of synonymous evolution will underestimate the rate of mutation (Sharp et al. 1988; Shields et al. 1988; Sharp and Li 1989; Li 1997). Given that previous reports have suggested that duplicate genes are expressed at particularly high levels in S. cerevisiae (Seoighe and Wolfe 1999), their rate of synonymous evolution should be lower than that of singletons even in the absence of mutational differences.

To overcome this complication, we computed the partial correlation coefficients between each of the three factors: codon bias (measured by the codon adaptation index [CAI] [Sharp and Li 1987] in *D. melanogaster)*, gene class (whether the representative pair was for a duplicate or singleton), and *K*_S _(between representative pairs). Our results, presented in Table 2, reveal that, as expected, representative pairs of S. cerevisiae duplicate genes have a lower *K*_S_ than representative pairs of singleton genes (Spearman Correlation column), but that this correlation disappears when we control for codon bias (Partial Correlation Coefficient column). Thus, in the case of *S. cerevisiae,* the higher codon bias of the slowly evolving representative pairs completely accounts for the differences in *K*_S_ between the two groups. For *C. elegans,* the *K*_S_ of the representative pairs for duplicate genes is in fact marginally higher than that for singleton genes, and this slight trend remains when codon bias is taken into account. Thus, mutational differences cannot account for the differences in the rate of protein evolution in either S. cerevisiae or C. elegans.

**Table 2:**
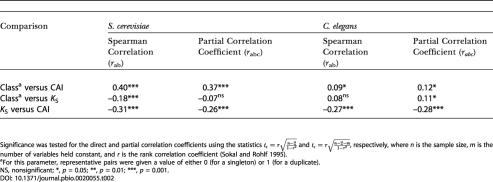
Correlation Coefficients and Partial Correlation Coefficients for the Three Factors Gene Class (Duplicate or Singleton), CAI (in D. melanogaster), and *K*_S _(of Representative Pairs)

Significance was tested for the direct and partial correlation coefficients using the statistics and , respectively, where *n* is the sample size, *m* is the number of variables held constant, and *r* is the rank correlation coefficient (Sokal and Rohlf 1995)

^a^For this parameter, representative pairs were given a value of either 0 (for a singleton) or 1 (for a duplicate)

NS, nonsignificant; *, *p* = 0.05; **, *p* = 0.01; ***, *p* = 0.001

### Codon Bias and the Rate of Evolution of Duplicate Genes

We can also use the level of codon bias to gain additional insight into the potential reasons for the generation and maintenance of duplicate copies of conserved genes. Codon bias is a proxy for the level of expression (Akashi 2001), while the level of expression is a good predictor of the rate of protein evolution (Pal et al. 2001; Krylov et al. 2003). To determine whether the reason for the slow evolution of duplication-prone genes is their higher level of expression, we performed a partial correlation analysis similar to the analysis of *K*_S_ above. Table 3 shows Spearman rank and partial rank correlations between pairs of the three variables gene class (singleton or duplicate study gene), CAI (in D. melanogaster), and *K*_A_ (of the representative pairs). This analysis revealed some important differences in how the duplication bias is generated in S. cerevisiae and C. elegans.

**Table 3:**
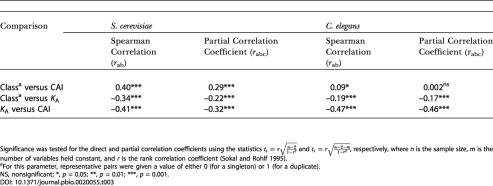
Correlation Coefficients and Partial Correlation Coefficients for the Three Factors Gene Class, CAI (in D. melanogaster), and *K*_A _(of Representative Pairs)

Significance was tested for the direct and partial correlation coefficients using the statistics and , respectively, where *n* is the sample size, *m* is the number of variables held constant, and *r* is the rank correlation coefficient (Sokal and Rohlf 1995)

^a^For this parameter, representative pairs were given a value of either 0 (for a singleton) or 1 (for a duplicate)

NS, nonsignificant; *, *p* = 0.05; **, *p* = 0.01; ***, *p* = 0.001

First, both direct and partial correlations for S. cerevisiae show that the CAI of the representative pairs of duplicates is greater than that of the representative pairs of singleton genes. This indicates that the genes leading to preserved duplicates in S. cerevisiae tend to be unusually highly expressed (*p* < 0.001). In contrast, for *C. elegans,* duplicate genes do not appear to be biased towards highly expressed genes (*p* > 0.1). This difference may reflect a disparity in the mutational generation, fixation, or preservation of duplicates in these two organisms. This analysis also reveals that when codon bias is held constant, the relationship between *K*_A_ and gene class persists in both organisms. In the case of C. elegans, the correlation coefficient between gene class and *K*_A_ remains nearly identical when CAI is held constant. For *S. cerevisiae,* the partial correlation coefficient between *K*_A_ and gene class does decrease when CAI is held constant (but remains highly significant), implying that the slower evolution of representative pairs of the duplicated genes in S. cerevisiae is partly mediated by preferential duplication of highly expressed genes. To validate these conclusions, we repeated the same analysis using CAI values in the study organisms rather than in D. melanogaster. This analysis revealed very similar results (data not shown).

### Time Uniformity of the Bias

**Figure 4:**
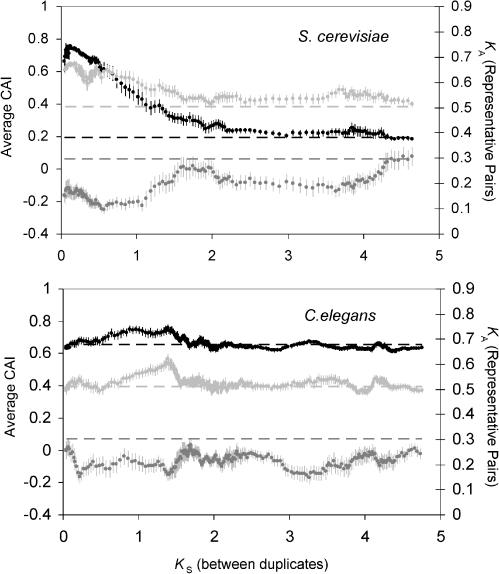
The Codon Bias and Rate of Evolution of Genes Leading to Duplicates over the Evolutionary History of S. cerevisiae and C. elegans For both S. cerevisiae (A) and C. elegans (B), moving averages of nonsynonymous substitutions per site (*K*_A_, in dark gray), codon bias in the study organism (measured with CAI, in black), and codon bias of the representative ortholog in D. melanogaster (CAI, in light gray) are plotted against the number of synonymous substitutions per site (*K*_S_) between duplicate pairs. The bin size is 15, and standard error bars are shown. Dashed lines represent the average CAI of singleton genes and the average *K*_A_ of representative pairs of singleton genes.

To determine whether conserved genes have been preferentially duplicated throughout the history of the S. cerevisiae and C. elegans lineages, we plotted the evolutionary rate of representative pairs and the average CAI (both in D. melanogaster and in the study organisms) for duplicate pairs of different age classes (where age is measured by *K*_S_ between the duplicate study genes) (Figure 4). While large *K*_S_ estimates are subject to a large amount of error (such that estimates of *K*_S_ above 2 are typically unreliable), this analysis captures the uniformity of the bias in these lineages. For both organisms, slowly evolving genes appear to have led to the duplicate genes in all age classes (covering hundreds of millions of years). For *C. elegans,* both the evolutionary rates of the representative pairs and their CAI values remain virtually constant for duplicated genes of all ages. In addition, the CAI values for the duplicate pairs of different ages in C. elegans are very similar to the CAI values for singletons—the only exception is a slight elevation in the CAI for duplicate pairs in the *K*_S_ range from 1 to 1.5. By contrast, the plot for S. cerevisiae reveals that young duplicate genes (*K*_S_ < 2.0) tend to have representative pairs with a lower *K*_A_ than those of older pairs, and this trend is paralleled by the elevated CAI of these young duplicate pairs.

**Figure 5:**
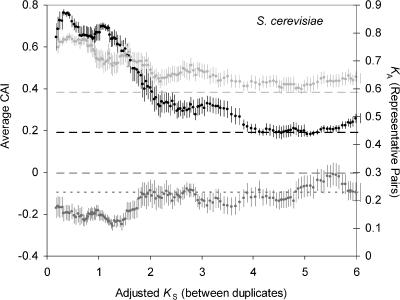
Correcting for Synonymous Substitutions Reveals That S. cerevisiae Genes That Have Recently Duplicated Have a Higher Codon Bias and Slower Rate of Evolution Than Those That Duplicated in the Ancient Past For duplicate genes in S. cerevisiae, moving averages of the number of nonsynonymous substitutions per nonsynonymous site of representative pairs (*K*_A_, in dark gray), the codon bias in S. cerevisiae (CAI, in black), and the codon bias of representative pairs in D. melanogaster (CAI, in light gray) are plotted against the adjusted number of synonymous substitutions per site (see Materials and Methods) between duplicate pairs. The bin size is 15, and standard error bars are shown. Lines with broad dashes show the respective averages for singleton genes in *S. cerevisiae,* and the line with short dashes shows the average *K*_A_ for representative pairs of duplicate genes in C. elegans.

A problem for interpreting this trend in S. cerevisiae is that duplicate pairs with a high codon bias are expected to have a depressed value of *K*_S_, as discussed above, and thus will appear younger than they really are. To overcome this problem, we corrected *K*_S_ estimates for S. cerevisiae genes based on their CAI using a simple approach recently developed for this species (see Materials and Methods) (A. Hirsh, H. Fraser, and D. Wall, personal communication). After correcting *K*_S_ estimates, the plots of *K*_A_ and CAI shift slightly (Figure 5), but the trends remain. We can further see that the duplicate pairs with the unusually high CAI and the unusually low *K*_A_ of the representative pairs have corrected *K*_S_ less than 2.0. It is intriguing that this age range matches the estimated time of the whole-genome duplication in the S. cerevisiae lineage (*K*_S_, approximately1.0; 80 million years ago) (Wolfe and Shields 1997; Pal et al. 2001). If the set of genes preserved after polyploidization in S. cerevisiae was biased towards highly expressed genes, this could explain the heterogeneity in both *K*_A_ and CAI and could explain why duplicate genes in C. elegans, an organism that has likely not undergone a whole-genome duplication, were not enriched for genes with a high level of expression. With respect to this hypothesis, it is interesting to note that for young duplicate genes (*K*_S_ < 2), *K*_A_ estimates for representative pairs of duplicate genes in S. cerevisiae are much lower than for duplicate genes in C. elegans, whereas for older duplicate genes (*K*_S_ > 2), the *K*_A_ estimates are roughly equivalent in both S. cerevisiae and C. elegans.

**Figure 6:**
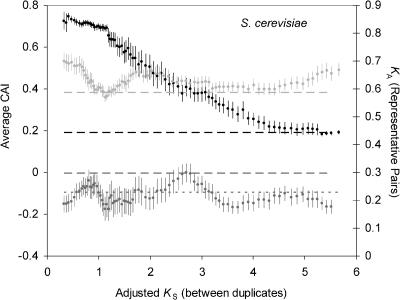
After Removing Ribosomal Genes, the Magnitude of the Bias towards the Slower Evolution of Duplicate Genes Is Similar in Both S. cerevisiae and C. elegans For nonribosomal duplicate genes in S. cerevisiae, moving averages of the number of nonsynonymous substitutions per nonsynonymous site of representative pairs (*K*_A_, in dark gray), the codon bias in S. cerevisiae (CAI, in black), and the codon bias of representative pairs in D. melanogaster (CAI, in light gray) are plotted against the adjusted number of synonymous substitutions per site (see Materials and Methods) between duplicate pairs. The bin size is 15, and standard error bars are shown. Lines with broad dashes show the respective averages for singleton genes in *S. cerevisiae,* and the line with short dashes shows the average *K*_A_ for representative pairs of duplicate genes in C. elegans.

Other studies have noted that ribosomal subunit proteins were particularly prolific in generating duplicate pairs via polyploidization in S. cerevisiae (Seoighe and Wolfe 1999). Indeed, these genes account for 49 of the duplicate pairs in our study. To determine whether this group is responsible for the depressed rates of evolution of young duplicate pairs, we plotted CAI and *K*_A_ versus *K*_S_ without ribosomal proteins (Figure 6). The plot reveals that without ribosomal proteins, young duplicate genes possess rates of evolution comparable to those of other age classes and more similar to the values found for duplicate genes in C. elegans. Thus, the overrepresentation of duplicate ribosomal proteins following the polyploidization event in S. cerevisiae appears to explain the low rates of evolution of young duplicate genes in this species. Even with these ribosomal genes removed, however, younger genes have much higher CAI values.

## Discussion

Most genome-scale studies of duplicate genes have focused either on the mechanisms of duplication or on the consequences of duplication at the molecular or organismal level. In this study we ask a different type of question: namely, which types of genes are more likely to duplicate than others? The method we use—identifying duplicate genes in one organism and obtaining evolutionary rate measurements from two outgroup species (see Figure 1)—allows us to compare the evolutionary rate of genes that have duplicated to that of those that have not. Importantly, it allows us to do this without confounding the effect the duplication itself has on the rate of molecular evolution (Lynch and Conery 2000; Kondrashov et al. 2002). Our data reveal that genes that have duplicated in the genomes of S. cerevisiae and C. elegans have much slower rates of amino acid substitution, as well as lower rates of insertion and deletion, on average than those that have remained in single copy.

To strengthen this conclusion, we tested several potential sources of error in our estimates of rates of evolution for the two classes of genes. We found that none of the potential complications—including the effect of duplication within the lineages of D. melanogaster and A. gambiae, duplications predating the split of the studied lineage and the outgroups, the especially slow evolution of multigene families, the operational definitions of duplicate and singleton genes, or the possibility of mutational differences—appear to affect our estimates of evolutionary rates of the two gene classes.

We have also attempted to ascertain whether conserved genes have been generating duplications in a persistent fashion or whether this bias was generated at a particular time in the history of the two studied genomes. Our analysis demonstrates that both lineages have experienced a consistent and very similar level of bias over hundreds of millions of years. In addition, there has been a recent duplication of particularly slowly evolving genes in the yeast genome, coinciding roughly with the time of the postulated genome duplication in this lineage. Importantly, the consistency of the pattern over such long evolutionary periods of time in such diverse lineages suggests that the preferential generation or retention of duplicates of slowly evolving genes might be a general feature of eukaryotic evolution.

Why do conserved, slowly evolving genes have a proclivity to generate duplicates? In order to answer this question, it is important to determine which of the three steps of duplication—mutation, fixation, or preservation—are responsible for this trend. As discussed above, both fixation and preservation have the potential to create a bias in the types of genes that duplicate. The step of fixation could generate a bias either because (1) many of the genes that are duplicated in a single individual are deleterious and thus are quickly removed from the population or (2) many of the duplicate genes that reach fixation in a population do so because of positive selection for the duplicate copy, rather than reaching fixation neutrally by genetic drift.

For the first mechanism to work, increases in the dosage of slowly evolving genes must be less likely to have deleterious consequences to organismal fitness than increases in the dosage of more rapidly evolving genes. Recent empirical work, however, has shown that the opposite might be true. In particular, data from yeast have shown that less dispensable, slowly evolving genes are more likely to be haploinsufficient than dispensable genes (Papp et al. 2003). This implies that changes in dosage of slowly evolving genes may have greater fitness consequences in general.

The second mechanism by which fixation may generate the bias is more tenable. This mechanism requires that many duplicate genes fix by positive selection and that duplicates of slowly evolving genes do so with higher likelihood. Examples from S. cerevisiae and bacteria (Romero and Palacios 1997; Brown et al. 1998; Dunham et al. 2002) support the possibility that duplications of genes can lead to beneficial increases in dosage and can be fixed by positive selection. One set of genes that may be especially likely to lead to beneficial increases in dosage following duplication are genes that are already required at high expression levels. It is interesting in this regard that many highly expressed genes have recently duplicated in S. cerevisiae (see Figure 5) (Seoighe and Wolfe 1999) and that the preferential duplication of genes with a high codon bias accounts partially for the bias that we observe in S. cerevisiae (see Table 3). While the preferential duplication of highly expressed genes is not observed for C. elegans, it is possible that duplications of slowly evolving genes are also likely to lead to beneficial increases in dosage for some other, yet unknown, reason.

The step of preservation also has the potential to generate the bias we observe since (1) many of the duplicate gene copies that arise in a population are lost quickly to nonfunctionalizing mutations (Lynch and Conery 2000) and (2) several models of duplicate gene preservation suggest that slowly evolving genes may have an increased likelihood of being preserved. In particular, these models predict the preferential preservation of genes with many *cis*-regulatory regions, expressed in many tissues (Lynch et al. 2001), or of genes that encode multidomain proteins (Gibson and Spring 1998; Stoltzfus 1999). Because the higher level and the greater breadth of expression, as well as the larger number of protein interactions, correlate with the slower rate of protein evolution (Duret and Mouchiroud 2000; Pal et al. 2001; Fraser et al. 2002), these models predict preferential preservation of slowly evolving genes.

If the step of preservation accounts for the slower evolution of duplicate genes, one prediction is that the rates of evolution of newly arisen gene duplicates should be higher than the rates of older gene duplicates and closer to the rates of evolution of singletons. Our data do not reveal any such trend for either S. cerevisiae or C. elegans (see Figure 4). The negative result, however, may simply reflect a lack of statistical power. The higher evolution rates of newly arisen gene duplicates should only be apparent for very young duplicate pairs. Indeed, the average half-life of a duplicate pair may be as short as 5 million years (Lynch and Conery 2000), corresponding to a *K*_S_ of approximately 0.05. There are very few such pairs in our dataset.

It is unclear whether fixation, preservation, or both of these steps together cause the bias towards the preferential duplication of slowly evolving genes. The relative importance of these two steps depends largely on the frequency with which duplicate genes are fixed by positive selection. If the vast majority of duplicate genes are initially redundant and fix by genetic drift, as assumed in many models of gene duplication (Ohno 1970; Force et al. 1999; Lynch and Force 2000; Lynch et al. 2001), fixation cannot explain the bias. If, on the other hand, duplicate genes often fix by positive selection (Kondrashov et al. 2002), the step of fixation may be dominant in generating the bias inthe types of genes that duplicate. The relative frequency with which duplicate genes fix because of positive selection and genetic drift remains to be established.

Beyond providing insight into the mechanisms of gene duplication, the bias has important consequences for the content of eukaryotic proteomes. If conserved, slowly evolving genes consistently generate preserved duplicate copies of themselves, proteomes will tend to become enriched for these genes over the course of evolution. This prediction is especially interesting in relationship to recent complementary work (Krylov et al. 2003) that shows that genes with a slow rate of evolution, a low dispensability, and a high level of expression are less likely to be lost over the course of evolution. Taken together, these two studies predict that slowly evolving genes should be the main sources of genes in eukaryotic genomes. It is also noteworthy that the two results are not independent. If slowly evolving genes are more likely to duplicate to form multigene families, then they should be less likely to be lost from a particular lineage, since this would entail the loss of many distinct genetic copies. The extent to which this effect explains the preferential loss of fast evolving genes remains to be determined.

The mere existence of this bias is very important for the interpretation of genomic-level studies of gene duplication. For example, some recent studies have argued that two general consequences of gene duplication are (1) an increased rate of evolution for the duplicated genes immediately following duplication (e.g., Kondrashov et al. 2002) and (2) increased functional redundancy at the genetic level (Gu et al. 2003). To make their arguments, both of these studies compare duplicate and singleton genes within a single organism under the assumption that the types of genes that duplicate are unbiased with respect to the molecular attribute of interest (note that a correction for this problem has been attempted before by separating genes into functional classes [e.g., Kondrashov et al. 2002]). The study presented here shows that this assumption is not valid. Duplicate genes are, in fact, a very biased set of genes, at least with respect to their rate of evolution. Interestingly, in the case of the studies just mentioned, the bias that we observed makes the conclusions conservative. Indeed, the bias that we observed may explain why other studies have failed to find the expected higher rate of evolution for genes that have recently undergone duplication (e.g., Kitami and Nadeau 2002). The preferential duplication of conserved genes, combined with the increased rate of evolution following duplication, may lead to no measurable difference in the rate of evolution between singleton and duplicate genes. In general, any genome-scale study that attempts to assess the effects of duplication on molecular evolution should consider the prior distribution of the molecular attributes of the genes that lead to duplicates.

## Materials and Methods

### 

#### Identification of duplicate and singleton genes and their orthologs

The gene and protein sequences of *S. cerevisiae, C. elegans, D. melanogaster,* and A. gambiae were downloaded from GenBank (Bethesda, Maryland, United States) at http://www.ncbi.nlm.nih.gov/Ftp/index.html. To identify duplicate and singleton genes, a reciprocal protein BLAST (Altschul et al. 1997) was performed on the proteomes of the two study organisms using default parameters and simple sequence filtering. Singleton genes were conservatively defined as those genes without a hit with an *E*-value of less than 0.1, following previous studies (Gu et al. 2003). Duplicate pairs in these genomes of S. cerevisiae and C. elegans were identified as reciprocal best hits with an *E*-value of less than 10^–10^ in both directions that could be aligned over greater than 60% of their lengths.

Orthologs were identified as reciprocal best BLAST hits between two organisms using the same criteria: *E*-values of less than 10^–10^ and alignable over greater than 60% of the gene lengths. In the case of duplicate pairs, the same criteria were used, except that both duplicates needed to hit the same gene in the outgroup species and the duplicate genes needed to be the top two best hits in the reciprocal blast. To identify representative pairs for each singleton and duplicate gene, we first identified an ortholog in D. melanogaster and then identified the ortholog of this gene in A. gambiae.

#### Obtaining *K*_A_ and indel measurements for representative pairs

To obtain the nucleotide alignments for each representative pair, we obtained the BLASTP alignment of the two orthologs, removed gaps in these alignments by trimming back from both ends of each gap until an anchor pair was found (following the method described in Conery and Lynch [2001]), and then replaced the amino acid alignment with the respective nucleotide sequence. Based on these alignments, we used the PAML software package (Yang 1997) to estimate the number of synonymous and nonsynonymous substitutions per site. The number of gaps per nucleotide length of each alignment was also recorded and used as a proxy for the number of indels that have occurred during the divergence of D. melanogaster and A. gambiae.

To test whether including duplicate pairs and singleton genes with representative pairs possessing paralogs in the D. melanogaster and A. gambiae lineages biased our results, we reanalyzed the distributions of nonsynonymous rates of evolution and number of indels after removing these genes. For both C. elegans and S. cerevisiae, we eliminated representative pairs with paralogs with a BLAST *E*-value less than 10^–10^ in either of the outgroup genomes (leaving 60 duplicates and 225 singletons and 48 duplicates and 530 singletons, respectively) and eliminated all representative pairs with paralogs with an *E*-value of less than 0.1 (leaving only 38 duplicates and 114 singletons and 29 duplicates and 318 singletons, respectively). Results from the reanalysis revealed significant trends similar to those found when using all representative pairs.

#### Obtaining CAI values and correcting *K*_S_

We obtained CAI values for genes in the D. melanogaster, S. cerevisiae, and C. elegans genomes using the program CodonW (available from ftp://molbiol.ox.ac.uk/Win95.codonW.zip; written by John Peden, now at Oxagen [www.oxagen.co.uk], and originally developed in the laboratory of Paul Sharp, Department of Genetics at the University of Nottingham, United Kingdom). The table used to calculate CAI for S. cerevisiae is the standard table included in the package. We obtained the appropriate codon usage tables for C. elegans and D. melanogaster from studies by Duret and Mouchiroud (1999) and Carbone et al. (2003), respectively.

For duplicate genes in *S. cerevisiae,* we used CAI values of each pair to help obtain a better relative estimate of their ages. This was necessary because duplicate pairs with a high codon bias effectively have fewer neutral synonymous sites, resulting in the gross underestimation of their age based on *K*_S_ alone (Sharp et al. 1988; Shields et al. 1988; Sharp and Li 1989; Li 1997). A recent study has shown that the number of synonymous substitutions expected for genes with a given codon bias in S. cerevisiae is given by *K*_S_ = *rt*(1 – *c*), where *r* is the rate of synonymous substitution in genes with no codon bias, *t* is time, and *c* is codon bias as measured by CAI (A. Hirsh, H. Fraser, and D. Wall, personal communication). Rearranging this equation yields the formula *K*_S_′ = *rt* = *K*_S_/(1 – *c*), which we used to obtain corrected estimates of the age of duplicate pairs in S. cerevisiae. No such correction was made for C. elegans genes because they were not shown to have a significantly higher codon bias than singleton genes and because no simple means of correction is presently known.

## Abbreviations

CAI: codon adaptation index
*K*_A_: the number of nonsynonymous substitutions per nonsynonymous site
*K*_S_: the number of synonymous substitutions per synonymous site
